# Effects of dance sports exercise on vestibular function and balance of children with sensorineural hearing loss; a randomized quasi-experimental trial

**DOI:** 10.3389/fped.2024.1426343

**Published:** 2024-08-15

**Authors:** Fang Hu, Xuan Qiu, Xinbo Wu, Xilong Wu, Han Li, Sukwon Kim

**Affiliations:** ^1^ College of Physical Education, Yichun University, Yichun, Jiangxi, China; ^2^ College of Sport, Exercise and Health Sciences, Loughborough University, Loughborough, United Kingdom; ^3^ Department of Physical Education, Jeonbuk National University, Jeonju, Republic of Korea; ^4^ College of Physical Education, Anhui Normal University, Wuhu, Anhui, China

**Keywords:** Latin dance, sensorineural hearing loss, vestibular function, balance, children

## Abstract

**Background:**

Sensorineural hearing loss (SNHL) children have difficulty living and limited movement due to impaired vestibular function and reduced balance ability.

**Objectives:**

The present study evaluated the effects of Latin dance training on the vestibular function and balance of SNHL children.

**Methods:**

Thirty SNHL children with no difference in vestibular function and balance ability were randomly divided into two groups, the Latin dance training group (LTG, *n* = 15) and the control group (CONG, *n* = 15). Vestibular function, timed eyes-closed static (ECS) and functional reach test (FRT) were measured before and after the intervention, and a two-way repeated-measures analysis of variance was performed.

**Results:**

After training, the vestibular function performance of LTG was higher than that of CONG (CONG: 16.425 ± 3.616 vs. LTG: 12.241 ± 2.610, *p* = 0.0411, ES = 1.3914), the left foot ECS performance of LTG was higher than that of CONG (CONG: 2.765 ± 0.872 vs. LTG: 4.688 ± 1.113, *p* = 0.0026, ES = 1.9857), the right foot ECS performance of LTG was higher than that of CONG (CONG: 3.113 ± 0.639 vs. LTG: 4.797 ± 1.071, *p* = 0.0137, ES = 2.01), the FRT performance of LTG was higher than that of CONG (CONG: 32.009 ± 6.134 vs. LTG: 43.797 ± 6.616, *p* = 0.0021, ES = 1.9135).

**Conclusions:**

After Latin dance training, SNHL children improved vestibular function and balance. The static balance ability of the left foot has been improved significantly than right foot.

## 1 Introduction

Listening and language are the main means of communication in everyday life. They are involved in almost all life tasks, such as providing information, communicating, and asking for help. Childhood hearing impairment is a significant public health problem in the world (1, 2). Symptoms of hearing impairment in children include sensorineural hearing loss (SNHL) and conductive hearing loss, which affects 1–6 in 1,000 children and is increasing (3–5).

Vestibular dysfunction are the most common type of injury in children with SNHL (6), some studies have found that up to 41%–85% of children with SNHL have vestibular system disease (7–10). Vestibular dysfunction is characterized by impaired spatial orientation and balance due to abnormalities in the vestibular system's functioning. Since the auditory and vestibular organs are closely related and the receptors for these organs are in the inner ear, if the cochlea, semicircular canal or the otolith organs is damaged, it can lead to abnormalities in the vestibular system related to postural balance (11–13), These structures are critical for providing information about head motion relative to gravitational forces, detecting both linear accelerations and head tilts, thereby playing a crucial role in maintaining equilibrium and spatial orientation. Due to these properties, SNHL children with bilateral severe vestibular impairment show delayed responses to rapid or complex tasks (14) and impaired balance and postural control (15, 16), additionally, vestibular dysfunction negatively affects cognitive development (17), executive function (18), working memory (19), and social-emotional behavior, therefore SNHL children experience difficulties with reading, writing, and study skills, as well as fatigue, avoidant behavior, low self-esteem, anxiety, and depression (17, 20–22).

At the same time, balance is a necessity for children to carry out activities of daily living. This kind of adjustment of body balance requires the coordination and integration of the visual system, proprioception system, vestibular system and the body's central nervous system in order to maintain body balance during movement reactions (23). Numerous studies have reported impaired balance (static or dynamic) in children with SNHL, who have lower motor performance, including balance and other motor skills, compared to children with normal hearing, which may be related to vestibular dysfunction caused by inner ear injury (24–27). Therefore, it is necessary to design an exercise program for children with SNHL that can help communication, body organ development, and balance.

Dancesports have become popular in the world as an Asian Indoor Games event. It is an aerobic fitness and entertainment sport that can cultivate your own temperament, promote psychological happiness, and is suitable for men, women, elder and children (28, 29). Among them, the main technical characteristics of Latin dance (includes dances such as the cha-cha-cha, rumba, samba, jive, and paso doble) are high-quality body posture control technology, rapid body center of gravity shifting technology and whole-body muscle rhythmic twisting technology (28, 30). Multiple studies have shown that long-term Latin dance training can help improve the body's cardiorespiratory endurance, muscle strength, speed, flexibility, balance and other physical fitness indicators (28, 31–34).

The following study was designed to explore whether a Latin dance intervention can improve vestibular function and balance in children with SNHL. The present study hypothesized that both vestibular function and postural balance would improve following the intervention.

## 2 Materials and methods

### 2.1 Participants

**Figure 1:**
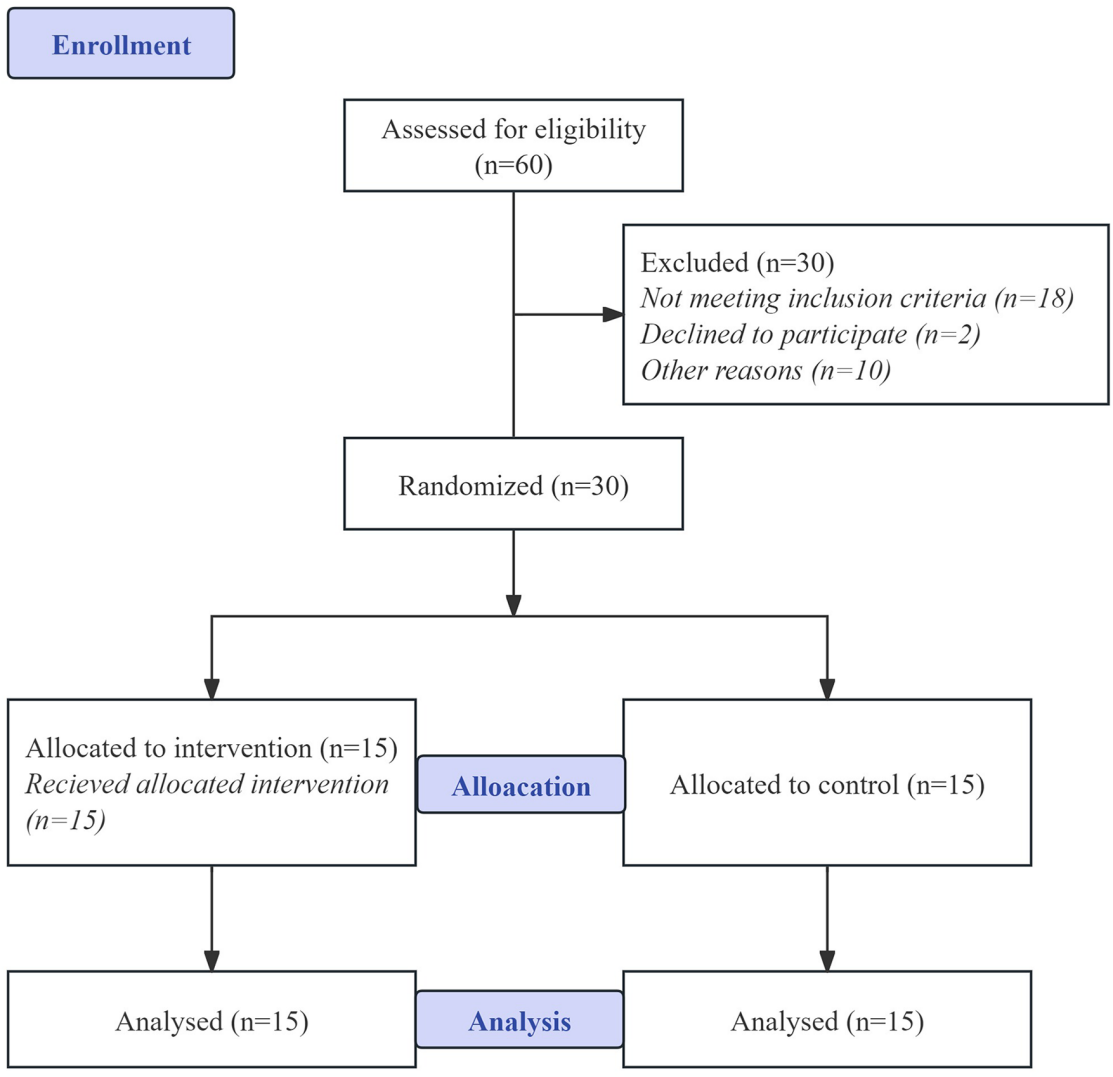
Study flowchart.

This cross-sectional study was conducted on thirty congenital SNHL children in special schools in Yichun, China, between September and December 2022. The participants, aged between 10 and 18 years old, were diagnosed with SNHL at the time of assessment, confirmed through audiogram results indicating a degree of hearing loss greater than 20 dB HL. Participants were excluded if they had purely conductive hearing loss, normal hearing (under 20 dB HL) on their most recent audiogram, other peripheral vestibular diseases, or had taken medication for vertigo or dizziness in the past month. Additionally, none of the participants had regularly engaged in sports activities or dance classes in the past two months, had a history of physical injuries, or exhibited cognitive impairments. Members of the research team utilized the deviation angle of vertical writing test (with deviations exceeding ten degrees considered abnormal and indicative of vestibular dysfunction) (35) and the Functional Movement Screen (FMS) (36) as benchmarks to assess the initial vestibular functions and balance abilities of the participants and randomly assigned thirty SNHL children into two groups (Latin Dance training group (LTG) and control group (CONG)). Figure 1 shows the progress of enrollment. There were no disparities in the baseline characteristics between the two groups before the intervention (Table 1). All participants signed informed consent forms approved by the University Ethics Committee (JBNU2022-01-004-002).

**Table 1:** Basic information of experimental subjects.

Group	Age	Height (cm)	Weight (kg)	FMS (score)
CONG (*n* = 15)	14.889 ± 2.208	151.81 ± 2.21	36.96 ± 1.21	10.45 ± 1.79
LTG (*n* = 15)	15.333 ± 2.121	152.25 ± 1.98	36.58 ± 2.59	10.15 ± 1.90
*P*	0.66	0.74	0.89	0.61

### 2.2 Intervention

From September to December 2022, the intervention focused on Cha-cha dance (a type of Latin dance) due to its easy-to-learn nature and suitability for the participants in terms of activity and exercise intensity. The participants attended sessions five times a week for 12 weeks and each session lasting 45 min with a 5 min break in between. The main content of training curriculum included basic steps of Cha-cha dance, single and double combination routines, dance music understanding exercises. Each course was divided into three stages: basic (weeks 1–2), consolidation (weeks 3–7), and improvement (weeks 8–12). To assess teaching effectiveness and quality of the teachers, the SNHL children performed a stage report after the whole training, which was affirmed and appreciated by the school leaders and parents.

### 2.3 Measurements

#### 2.3.1 Vestibular functions

##### 2.3.1.1 The deviation angle of vertical writing test

**Figure 2:**
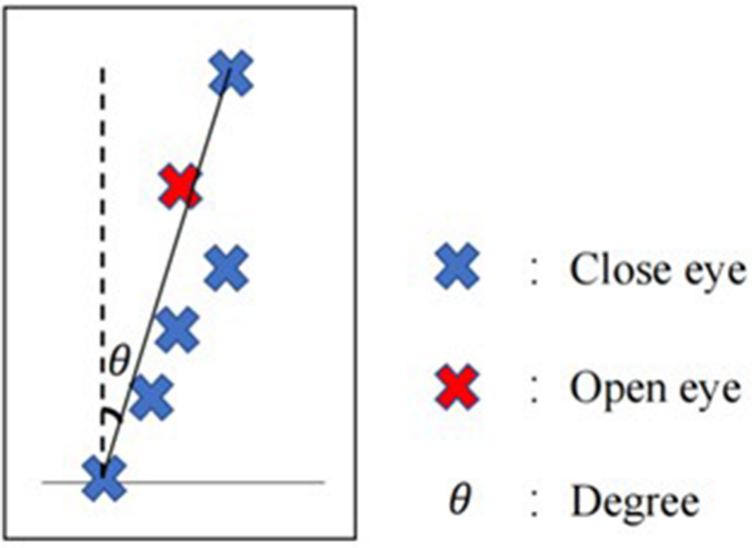
Vertical writing test.

Participants were asked to face a wall with a blank sheet of paper affixed to it. They were asked to write an “X” with eyes open as the true vertical direction, and then write “X” 5 times in a row with eyes closed. The tester uses a protractor to measure the angular difference between the different written letter and the true vertical direction. The inclined angle between the uppermost “X” center and the lowermost “X” center and the vertical line indicated the severity of vestibular dysfunction (Figure 2). Enhanced vestibular function should be associated with smaller angles. They were tested 3 times and the average angle was used (37).

#### 2.3.2 Static balance ability

##### 2.3.2.1 Timed eyes-closed static (ECS)

**Figure 3:**
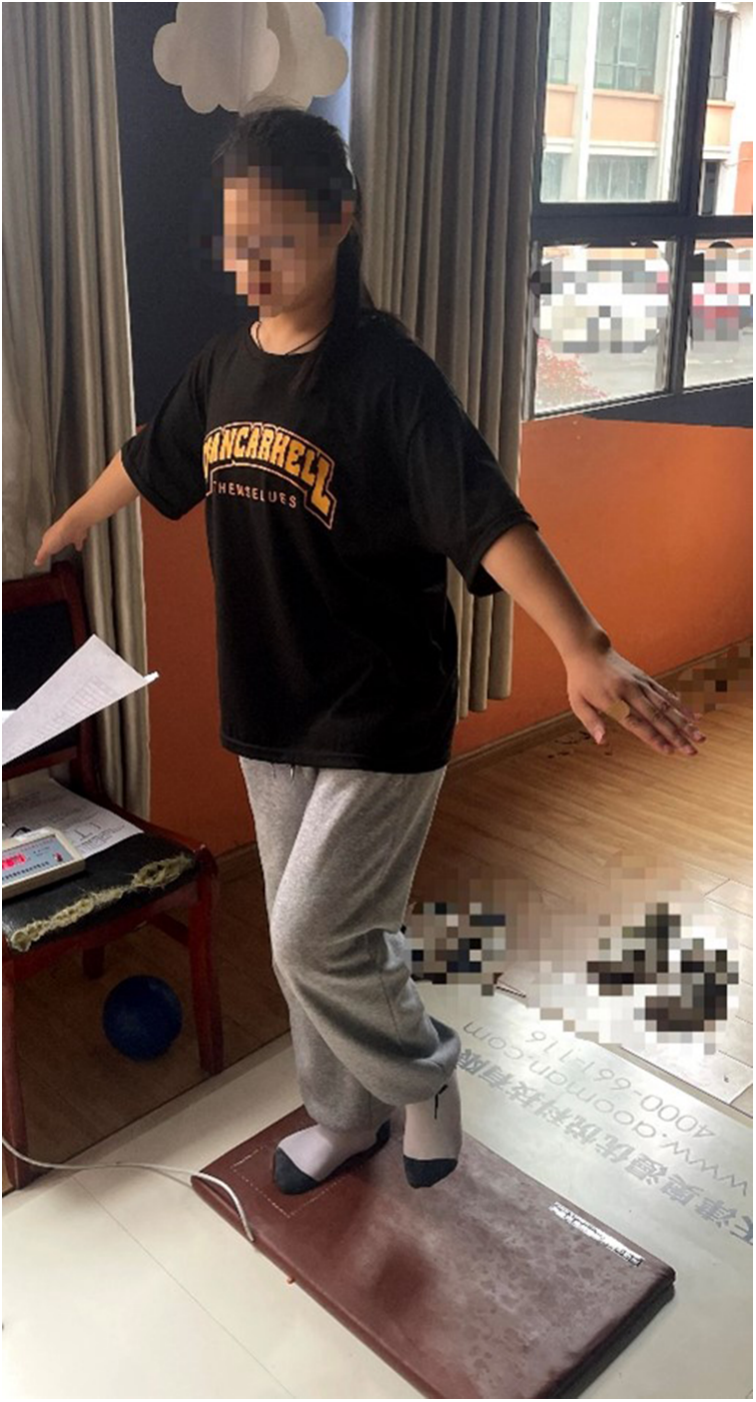
Timed eyes-closed static (ECS).

Adequate test-retest reliability for timed static unipedal balance has been reported in both children and adults (38, 39). According to the study: Timed eyes-closed static (ECS) and eyes-closed dynamic (ECD) balance (using an Airex Balance Pad for base of support), with a 180 sec maximum for each test, were appropriate clinical balance measures for adolescents (40). In this study, participants were tested using the single-foot balance test apparatus with eyes closed. They were asked to take off their shoes and socks to stand on one foot in the test area of the balance pad. The display screen connected to the balance pad started timing and the timing ended when they were no longer able to stand on one foot. Their left and right feet were tested separately, starting with the left foot, followed by the right foot, and the average of three consecutive tests was taken (Figure 3).

#### 2.3.3 Dynamic balance ability

##### 2.3.3.1 Functional reach test (FRT)

**Figure 4:**
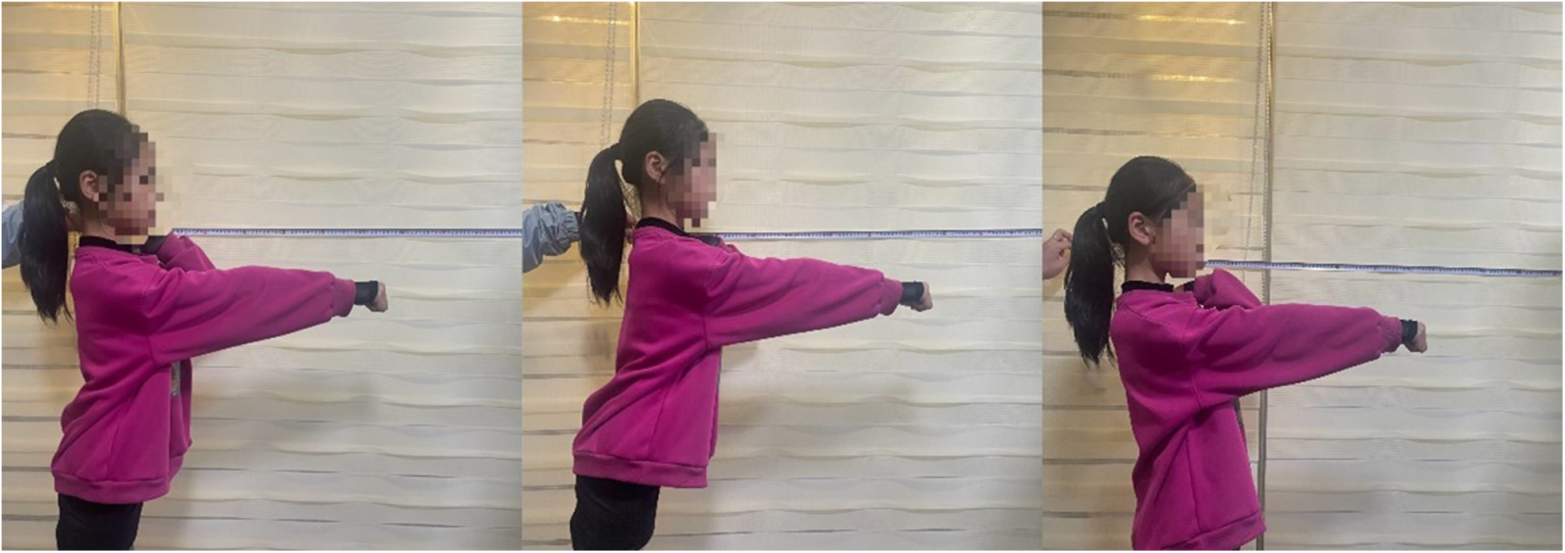
FRT in front and rear direction.

**Figure 5:**
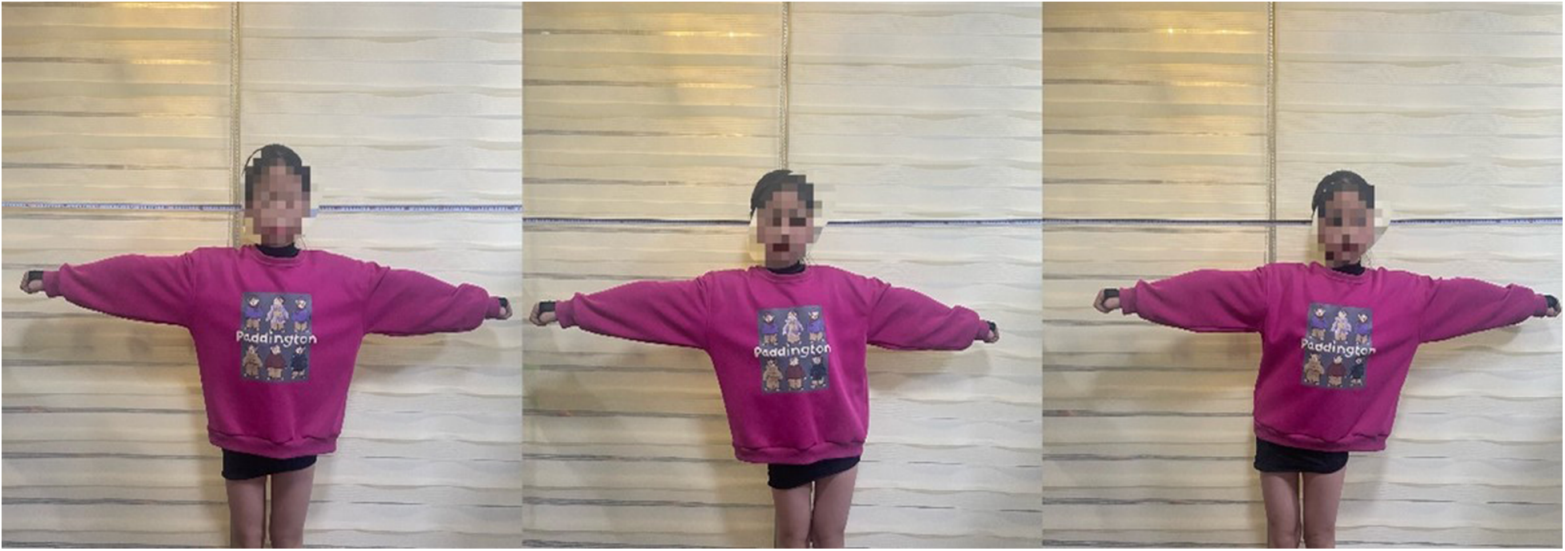
FRT left and right direction.

The participants stood with feet together, with the sagittal plane parallel to the wall (distance marks are painted on the wall), about ten centimeters away from the wall, with the dominant side leaning against the wall, shoulders flexed at 90°, elbows extended and fists made, maintaining body lines, and recorded The position of the metacarpophalangeal joint (marker 1), and then extend as far forward as possible with the ankle joint as the axis without taking a step, record the position of the farthest metacarpophalangeal joint (marker 2), and measure the distance between the two marks (Figure 4); then the participants restores vertical position, record the position of the shoulder joint (Mark 3), tilt as far back as possible without taking a step back, record the farthest position reached by the shoulder joint tilting backward (Mark 4), and measure the distance between the two marks; the participants’ back stand against the wall with your feet together, abduct your upper limbs to a horizontal position, maintain the body's line of force, and tilt as far as possible to the left and right, and measure the displacement distance of the palm joints in the same way as above (Figure 5). The participants were tested twice in four directions, and the average of the above eight values for each subject was taken as the FRT value, and the recording unit was centimeters (41, 42).

### 2.4 Statistical methods

Statistical analyses were performed using GraphPad Prism 9.2.0 (GraphPad Software, La Jolla, CA, United States). To achieve research objectives (i.e., evaluation of a Latin dance intervention to improve vestibular function and balance in children with SNHL), a t-test was performed to analyze the pre-intervention variables. Data were expressed as mean ± SD, and statistical significance was accepted as *p* < 0.05. Variables were analyzed using two-way repeated-measures ANOVA, followed by a Šidák's multiple comparisons, the factors were intervention conditions (CONG/LTG) and time (pre/post). Cohen's d effect size (ES) was reported (ES > 0.2, small; >0.5, moderate; >0.8, large) when statistical differences were found between conditions.

## 3 Results

### 3.1 Comparison of baseline features before intervention

In all measures of variables, there was no significant difference between LTG and CONG (*p* > 0.05) (Table 2).

**Table 2:** Subjects’ variables characteristics during the baseline test (pre- intervention).

Variable	CONG(*n* = 15)	LTG(*n* = 15)	*P*
BMI (kg/m^2^)	18.011 ± 4.199	17.908 ± 3.939	0.95
Vestibular functions(°)	16.345 ± 3.569	16.692 ± 3.093	0.58
Left foot ECS(s)	2.597 ± 0.846	2.464 ± 0.775	0.66
Right foot ECS(s)	3.093 ± 0.621	3.117 ± 1.219	0.95
FRT(cm)	32.154 ± 6.332	33.127 ± 6.316	0.68

### 3.2 Effect of intervention

Results of vestibular functions are depicted in Table 3. Following ANOVA, a main time effect was found (*p* < 0.01). The vestibular functions performance of LTG was higher than that of CONG after training (CONG: 16.425 ± 3.616 vs. LTG: 12.241 ± 2.610, *p* = 0.0411, ES = 1.3914). At the same time, it was found that through Latin dance training, there was a significant interaction between time × group (*p* < 0.01). Vestibular functions indicators were similar at baseline and significant improved after training.

**Table 3:** Results of indicators pre- and post- intervention.

Variables	Groups	Pre-test	Post-test	Interaction
BMI (kg/m^2^)	CONG	18.011 ± 4.199	18.271 ± 3.454	
	LTG	17.908 ± 3.939	18.444 ± 3.726	
Vestibular functions(°)	CONG	16.345 ± 3.569	16.425 ± 3.616	<0.01
	LTG	16.692 ± 3.093	12.241 ± 2.610*	
Left foot ECS(s)	CONG	2.597 ± 0.846	2.765 ± 0.872	<0.01
	LTG	2.464 ± 0.775	4.688 ± 1.113**	
Right foot ECS(s)	CONG	3.093 ± 0.621	3.113 ± 0.639	<0.01
	LTG	3.117 ± 1.219	4.797 ± 1.071**	
FRT(cm)	CONG	32.154 ± 6.332	32.009 ± 6.134	<0.01
	LTG	33.127 ± 6.316	43.797 ± 6.616**	

Two-way repeated measures ANOVA with a Šidák's *post-hoc* test for all.

Data are presented as the mean ± sd (*n* = 30).

* *p* < 0.05 vs. CONG.

** *p* < 0.01 vs. CONG.

Results of left foot ECS are depicted in Table 3. Following ANOVA, a main time effect was found (*p* < 0.01). The left foot ECS performance of LTG was higher than that of CONG after training (CONG: 2.765 ± 0.872 vs. LTG: 4.688 ± 1.113, *p* = 0.0026, ES = 1.9857). At the same time, it was found that through Latin dance training, there was a significant interaction between time × group (*p* < 0.01). Left foot ECS indicators were similar at baseline and significant improved after training.

Results of right foot ECS are depicted in Table 3. Following ANOVA, a main time effect was found (*p* < 0.01). The right foot ECS performance of LTG is higher than that of CONG after training (CONG: 3.113 ± 0.639 vs. LTG: 4.797 ± 1.071, *p* = 0.0137, ES = 2.01). At the same time, it was found that through Latin dance training, there was a significant interaction between time × group (*p* < 0.01). Right foot ECS indicators were similar at baseline and significant improved after training.

Results of FRT are depicted in Table 3. Following ANOVA, a main time effect was found (*p* < 0.01). The FRT performance of LTG was higher than that of CONG after training (CONG: 32.009 ± 6.134 vs. LTG: 43.797 ± 6.616, *p* = 0.0021, ES = 1.9135). At the same time, it was found that through Latin dance training, there was a significant interaction between time × group (*p* < 0.01). FRT indicators were similar at baseline and significant improved after training.

In addition, we also found an interesting phenomenon. In the ECS test, both the left and right feet had improved compared to before the intervention, but the ECS of the left foot seems to have improved even more (CONG: 2.765 ± 0.872 vs. LTG: 4.688 ± 1.113). At the same time, after the intervention, concurrent enhancements in the ECS indicators were more balanced in both feet (Left: 4.688 ± 1.113 vs. Right: 4.797 ± 1.071).

In summary, after 12 weeks of Latin dance training intervention, although the body shape of SNHL children did not improve significantly before and after the intervention, their vestibular function, static balance ability (left and right feet) and dynamic balance ability were significantly improved, and in terms of static balance, the left foot improved more than the right foot.

## 4 Discussion

This study aimed to evaluate the effect of 12 weeks of Latin dance training on the vestibular function and balance ability of SNHL children. The results of the study showed that 12 weeks of Latin dance training effectively improved the vestibular function and balance ability of SNHL children, and consistent with the research results of Kamel, RM (43), Tuncer, D (44), Soori, Z (45) et al.

Postural responses and the ability to maintain body balance in stability and in motion is a coordination of visual, tactile, auditory and intrinsic receptors (46), Vestibular receptors receive stimuli from head position, and plays an important role in basic motor function responses such as reflexes and body position maintenance (47). In particular, the organs related to sound are closely related to the vestibular system, and the inner ear transmits information to the brainstem through cranial nerve No. 8. Due to the close neuroanatomical linkage of this system, damage to the cochlea, semicircular canals, or both induces vestibular dysfunction, resulting in impaired balance (11, 12).

The changes in vestibular function tests were obvious before and after training, and the test results after LTG group are significantly improved compared with the test results before LTG and the test results after CONG. Between 30% and 85% of deaf children with moderate to severe hearing loss reported loss of vestibular function, according to a pilot study (48, 49). The function of the vestibular system is positively related to the postural balance of the body. In this study, the single-leg support movement often used in Latin dance training (29), and perform forward and backward movements while standing opposite to the dance partner. At the same time, in order to maintain this upright standing posture and body balance, participants need to continue to use the muscles of the spine and lower limbs (50), and the function of mobilizing muscles through nerves is strengthened (29), thereby improving the vestibular nerve function of SNHL students.

The static balance ability (left and right feet) changed significantly before and after training. The test results after the LTG group were significantly improved compared with the test results before LTG and the test results after CONG. It is difficult for SNHL children to maintain correct body posture or balance in daily activities. Regular physical activity improves stability and balance (51), specifically, Latin dance training can effectively improve the balance of the body (29, 52), and it has been confirmed through the training of Cha-cha dance intervention method. In each Cha-cha dance training class, the teacher arranged dance standing posture exercises and maintain stability, which was very important for the high requirements of body control and balance stability in dance operation. Since Cha-cha dance is a dance with a medium-speed music rhythm, dancers must perform short fast movements and delayed slow movements under this rhythm to reflect the beauty of the dance, this repeated switching may enhance the inhibition of neural differentiation and strengthen proprioception (53), thereby improving the static balance ability of the feet. Static balance appeared to improve more on the left foot than before the intervention, while concurrent enhancement was more balanced on both feet after the intervention. This may be caused by the unique biomechanical characteristics and dance movement characteristics of Cha-cha dance. The basic dance steps of Cha-cha dance are mostly symmetrical movements (symmetry of direction, symmetry of distance, symmetry of force), it is known that the right leg is usually the dominant leg for most individuals. In a task performance environment, the right leg is biased when standing, supporting weight, and coordinating movements. Over time, the left side becomes relatively weak, and the brain continues to strengthen the dominance mode of the right side (54). When the left and right feet bear the same training load and intensity, and most people's left limb is not dominant, the left foot may be better than the right foot and concurrent enhancement was more balanced. The results of this experimental study verify the above model and are consistent with the results of a series of previous studies by the research team (29).

The dynamic balance ability changed significantly before and after training. The test results after the LTG group were significantly improved compared with the test results before LTG and the test results after CONG. Through long-term basic movement training of Cha-cha dance, the muscles of the lower limbs are stimulated, which strengthens the stability of the ankles and knees of SNHL children and helps to develop the flexibility of the joints (28, 55). Dance tasks require dancers to lengthen their body lines to show the beauty of dance. Long-term repetitive movement training reduces muscle tension and effectively improves flexibility (56, 57). At the same time, the proper function of ankle joint (i.e., ankle strategy) is important in means of postural balance (58). Cha-cha Dance's repetitive front-back and left-right symmetrical dance steps, as well as plantar and dorsal (anterior-posterior) flexion movements, and medio-lateral movements (inversion-eversion), and thus contributes to the improvement of the ankle muscle strength. Due to the long-term repetition of this exercise, the elastic muscle strength of the ankle joint increases, has resulted in shorter contact with the ground, which further caused quicker body weight transfer and better control in the change of movement direction. Proprioception mechanisms play essential roles in regulating balance by way of neuromuscular control (59). Dance is an art form that combines aesthetic characteristic with physical abilities such as postural control and static and dynamic balance. Therefore, the test indicators of FRT of SNHL children have been significantly improved after training, as well as their dynamic balance ability.

## 5 Conclusions

After 12 weeks of Latin dance training for SNHL children, vestibular function and balance ability have been effectively improved, compared with the right foot, the static balance ability of the left foot has been improved more significantly. Therefore, for SNHL children with reduced physical activity due to hearing impairment, Latin dance is a suitable exercise program that can improve motor function execution and serves as a sports rehabilitation program and school physical education course. In this study, due to China's COVID-19 management policy and lack of experimental equipment, there were fewer participants during the experiment, and the measurement of balance ability was slightly single. Therefore, this experiment will conduct a follow-up in-depth study on the intervention of Dancesports on the balance ability of SNHL children and explore its specific mechanism of action.

## Data Availability

The raw data supporting the conclusions of this article will be made available by the authors, without undue reservation.
